# Perceived and Received Social Support and Illness Acceptance Among Breast Cancer Patients: The Serial Mediation of Meaning-Making and Fear of Recurrence

**DOI:** 10.1093/abm/kaad067

**Published:** 2023-12-22

**Authors:** Dariusz Krok, Ewa Telka, Dagna Kocur

**Affiliations:** Institute of Psychology, Faculty of Social Sciences, Opole University, Opole, Poland; Department of Radiotherapy, Maria Sklodowska-Curie National Research Institute of Oncology, Gliwice Branch, Gliwice, Poland; Institute of Psychology, Faculty of Social Sciences, University of Silesia in Katowice, Katowice, Poland

**Keywords:** Social support, Illness acceptance, Meaning-making, Breast cancer patients, Fear of recurrence

## Abstract

**Background and Purpose:**

The literature indicates connections between social support and acceptance of a personal diagnosis of breast cancer, but these relationships are likely to be mediated due to numerous connections between social support and illness acceptance with meaning-making and fear of recurrence. We decided to investigate whether meaning-making and fear of recurrence serially mediated the relationship between perceived and received social support and illness acceptance.

**Methods:**

The current research examined 246 adult women patients with a breast cancer diagnosis who were undergoing radiotherapy/chemotherapy treatment in oncological hospitals. This cross-sectional study based on a mediational model was reported according to the STROBE checklist.

**Results:**

Our results supported the mediational model in which meaning-making and fear of recurrence serially mediated the relationship of both perceived and received social support with illness acceptance. Furthermore, the mediating power of meaning-making was more significant than the fear of recurrence.

**Conclusion:**

Meaning structures and anxiety played an important mediating role in breast cancer patients. Finding additional meaning and goals and experiencing lower fear enables the patients to effectively make use of social support and accept their illness.

**Practice implications:**

The current study identified factors that increase illness acceptance among breast cancer patients as it showed that patients can gain a greater understanding of the nature of their illness by becoming more aware of their own goals and values and reduce anxiety by learning about their current state of the illness.

## Teaser

Patients with breast cancer who received more social support had a better understanding of their illness and lower fear of recurrence. Women with breast cancer characterized by a sense of purpose and optimism experienced lower anxiety about a potential relapse of the disease.

## Introduction

Breast cancer is the most common cancer in women in the world [1]. The hardships of the cancer experience are considered potentially traumatic events associated with psychological effects [2]. Cancer has a psychologically difficult nature, among other things, because of the risk of recurrence and concerns related to the latter [3, 4]. However, factors such as social support and meaning-making can be very supportive in adjustment to illness [5, 6]. All this together can translate into greater acceptance of the disease, which helps in the recovery process [7, 8]. Therefore, we decided to investigate whether meaning-making and fear of recurrence serially mediated the relationship between perceived and received social support and illness acceptance.

### Associations Between Social Support and Illness Acceptance

*Social support* is one of the most critical features for coping with chronic, serious illness, especially cancer. It is very important for psychological functioning: For instance, social support is connected with lower levels of depression and anxiety [9]. Moreover, perceived and available instrumental support is also positively connected with quality of life [10]. Social support is also important in breast cancer patients, among other things, for predicting emotional well-being. In this group, social support influences coping strategies. Greater social support is positively connected with positive reframing and negatively with self-blame [5]. However, it is also important on a long-term scale. Received emotional support, such as reassuring, comforting, and problem-solving, in the months after the diagnosis significantly predicted posttraumatic growth even 8 years after the diagnosis [11]. Moreover, social support is essential not only for better psychological functioning, but also for physical functioning [12]. Meta-analyses conducted by Pinquart and Duberstein show that having high levels of perceived social support was associated with a decrease in relative risk of mortality for 25% of cancer survivors [12]. Additionally, reappraisal, one of the outcomes of social support, can indirectly lead to the creation of new meanings and assist in viewing the situation from an alternative vantage point [13].

*Illness acceptance* is also significant in struggling with a chronic disease, becoming a starting point for making choices essential for the treatment. Notably, illness acceptance is both connected with greater trust in doctors and treatment methods and with active participation in therapy [7, 8]. Reaching illness acceptance is a process part and parcel to psychological adjustment to chronic illness [14]. Previous research has shown that greater acceptance of an illness is associated with a sense of coherence, improved quality of life, reduced distress, lower levels of anxiety and depressive symptoms, and/or less perceived stress [10, 15–17]. On the other hand, a lack of illness acceptance or a low level of the latter is associated with distress, depressive symptoms, anxiety symptoms, greater pain, worse sleep, and social isolation [17, 18]. However, high, multidimensional pain makes it difficult to accept a personal disease diagnosis [19].

Importantly, social support and acceptance of an illness are interconnected [14, 20, 21]. Greater social support helps patients accept their illness [16]. For instance, social support correlated with acceptance-based strategies and acceptance of emotion, which were connected with illness acceptance; in addition, acceptance-based strategies and social support significantly mediated the relationship between benefit-finding and depression [22]. The mental adjustment to cancer is connected with acceptance of the illness [21].

Research conducted among cancer patients shows positive correlations between social relationships and acceptance of the illness [20]. Moreover, patients who lived alone had significantly lower acceptance of the illness [20]. In line with that are results obtained by Czerw et al. [23], where married individuals had a higher average acceptance of the illness than patients who declared themselves single.

To summarize, many studies indicate correlations between social support and illness acceptance, but none show their potential mediators. Further investigation is needed to reveal potential mediational links between these variables.

### Meaning-Making and Fear of Recurrence as Potential Mediators in Chronic Diseases

Confrontation with a life-threatening situation or disease can evoke *existential distress*, which is the experience of life with little or no meaning [24]. Thus, the possibility of death and an uncertain prognosis propels some cancer patients to question the meaning and purpose of life, and the answers tend to vary. There have been groups of cancer survivors who succeeded in meaning-making efforts, sometimes experiencing even more meaning in life than before the diagnosis [25]. Some of them experienced enhanced meaning after cancer through relationships, experiences, resilience, goal orientation, and leaving a legacy [26]. However, there is also a group of cancer survivors who struggle with meaning-making, have difficulties in this territory, and keep looking for meaning [27]. Sometimes, they experience a loss of meaning in their lives through experiences, social roles, relationships, and uncertainties about the future [26]. The above-mentioned relationships can be more deeply understood within the meaning-making model developed by Park [28]. The model makes an essential distinction between meaning-making efforts (e.g., automatic vs. deliberate processes or assimilation versus accommodation processes) and the meanings made as a result of such efforts (e.g., acceptance, perceptions of growth, integration of the stressful experience into identity, or reappraised meaning of the stressor). Such outcomes increase general acceptance of the situation and acceptance of cancer as a permanent element of one’s life, translating into less fear of cancer recurrence. Therefore, it is very likely that the overall level of illness acceptance in cancer patients will depend not only on social support, but also on the serial impact of meaning-making and fear of recurrence, as the latter may undermine the former in the illness situation. Considering the general framework through which cancer patients try to reorganize their lives, meaning-making and fear of recurrence may constitute significant serial mediators between social support and illness acceptance.

*Fear of cancer recurrence* and worries about disease progression are prevalent among cancer patients, especially in cases of breast cancer [3, 4]. Up to a quarter or even one-third of women with breast cancer experience moderate to high fear of disease progression [3, 4]. Such concerns may persist for many years for long-term cancer survivors and significantly affect the functioning of patients. Patients who experience more fear of cancer recurrence have a lower quality of life, even 6 years after the diagnosis [29], and in other studies, they have had higher emotional/psychological distress, cancer-specific distress, and/or depressive and anxiety symptoms [17]. Worrying about cancer progression naturally reduces one’s quality of life, but higher perceived social support can counteract it [30]. Additionally, socially isolated patients or patients with lower social support are at greater risk of experiencing fear of recurrence [31, 32].

The mediating function of meaning-making and fear of recurrence can be more clearly understood within the broader framework of stress and coping theory proposed by Park and Folkman [33]. *Meaning-making* is described as a psychological process that elucidates how people construe traumatic and potentially destructive incidents to achieve a coherent vision of life. As the meaning-making process consists of cognitive attempts to perceive, interpret, and evaluate personal experiences and events, it enables individuals to constructively cope with fear and anxiety. Theoretical concepts and results of empirical studies indicate that people perform a number of cognitive operations to understand the difficult and stressful experiences they deal with [28, 34, 35]. The process makes it possible to adaptively adjust by eliminating discrepancies in meanings through the reinterpretation and modification of existing beliefs [28, 35]. As a result of the cognitive actions taking place, an individual can develop a satisfactory system of meanings concerning specific events or experiences.

Research confirms the mediating value of meaning-making and fear of recurrence in chronic diseases [6, 36, 37]. *Life meaningfulness* was found to mediate between positive reframing and psychological well-being [6] and between affect and psychological well-being [36]. Moreover, meaning-making during cognitive behavioral therapy partially mediated between anxiety symptoms and depressive symptoms before and after treatment [37]. Fear of recurrence also mediated between fatalism and depressive and anxiety symptoms in women with breast cancer [38] and between external (e.g., contact with health professionals) and internal cues (e.g., feeling sick) and limited planning for the future [39].

## Methods

### Aim and Objective

A recent literature review implies connections between social support and acceptance of the illness [20, 21]. However, these relationships are likely mediated. Perceived social support also predicts patients’ tendency to consider cancer as a challenge, which could be connected with better meaning-making and, consequently, decrease the fear of cancer recurrence [31, 40]. Research suggests that social support and meaning-making are associated [5]. According to the buffering model of social support [13], social support may indirectly translate into meaning-making, among others, thanks to reappraisal. For example, talking to another person can help one see the situation from a different perspective. Moreover, according to the meaning-making model [28], meanings made, which refer to the products of meaning-making processes, such as the integration of the stressful experience into identity and the reappraised meaning of the stressor, may contribute to less anxiety, less fear of recurrence, and in turn, to more acceptance of the illness [28]. Additionally, according to the model of Simonelli et al. [41], appraisal involving creating new meaning can reduce fear of recurrence. Last but not least, the Family model of predictors of fear of recurrence also highlights the importance of social support for meaning-making, which translates into less fear of recurrence [42].

**Fig. 1.**
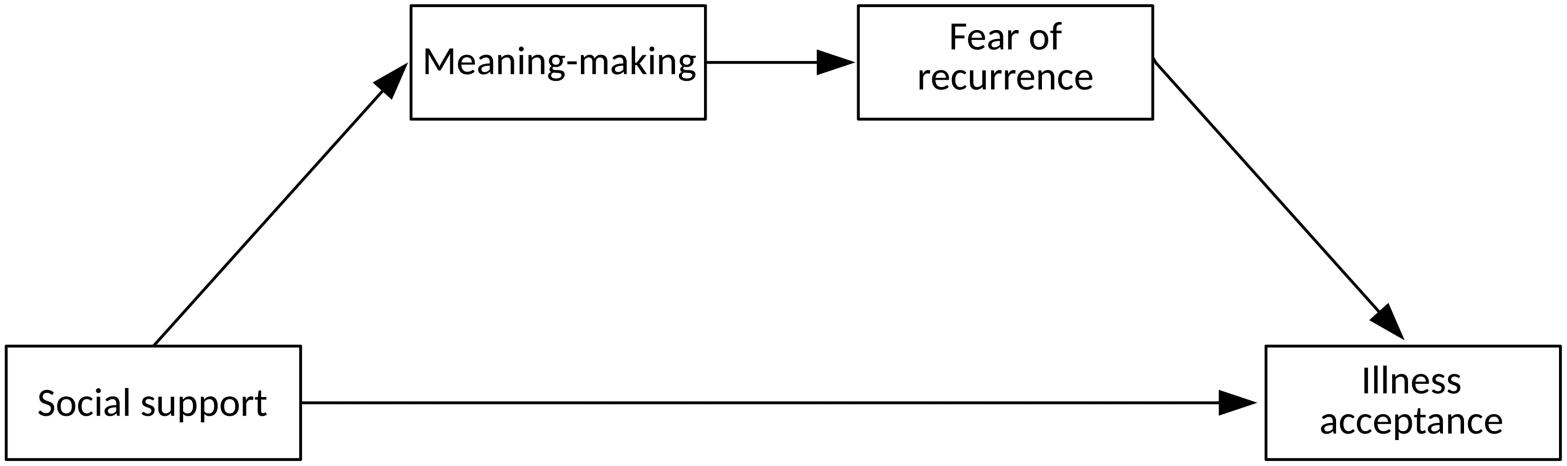
A model of the serial mediation of meaning-making and fear of recurrence in associations between social support and illness acceptance

To our knowledge, no one has examined mediational associations among perceived and received social support, illness acceptance, meaning-making, and fear of recurrence among breast cancer patients. Therefore, the aim of this study is to investigate whether meaning-making and fear of recurrence serially mediate the relationship between perceived and received social support and illness acceptance (Fig. 1). Based on both the meaning-making model and previous research, we formulated three hypotheses: (H1) Higher levels of perceived and received social support will be positively related to meaning-making and negatively related to fear of recurrence; (H2) higher meaning-making and lower fear of recurrence will be positively related to higher illness acceptance; and (H3) meaning-making and fear of recurrence will serially mediate the relationship between perceived and received social support and illness acceptance; more specifically, the character of the mediation will consist in higher meaning-making being related to lower fear of recurrence.

### Procedure

Initially, 297 patients were invited to participate in the study, whereas 31 declined to participate and 20 were not eligible due to unpredicted health conditions (e.g., seasonal flu, COVID-19 infections, malaise, and high blood pressure). A final sample of 246 participated in the study. The participation ratio was 83.11%. The participants were approached personally by research assistants while undergoing medical treatment in two university hospitals—the Maria Sklodowska-Curie National Research Institute of Oncology in Gliwice and the Voivodship Hospital in Opole—between September 2020 and June 2022. Participants were provided with an explanation of the study and given a set of questionnaires in a closed envelope to be completed within 2 weeks. Upon study completion, participants were invited for debriefing. The University of Opole Institutional Ethics Committee approved the study (approval number 2/2020). This study was reported according to the STROBE checklist.

### Participants

A total of 246 participants took part in our study. Eligible participants were adult women patients with a diagnosis of breast cancer who were undergoing radiotherapy/chemotherapy treatment in oncological hospitals. Criteria for inclusion were the following: (a) confirmed diagnosis of breast cancer (Stages I–III), (b) cognitive capacity to fill in questionnaires, and (c) positive physiological reactions to the treatment. Criteria for exclusion were the following: (a) cognitive deficiencies (e.g., serious memory problems, medical history of psychiatric disorders), (b) palliative treatment, (c) metastases to areas other than breasts, or (d) other serious medical conditions that could modify responses (e.g., cardiovascular problems, poor eyesight). The data were collected in Poland, and the participants were not paid for study participation.

### Measures

#### Social support

Two subscales of perceived and received social support from the Berlin Social Support Scales (BSSS) by Schulz and Schwarzer were used to measure domains of social support [43]. The BSSS was adapted and validated in Polish by Łuszczyńska et al. [44]. The subscales measure both cognitive and behavioral aspects of social support. The perceived social support scale comprises 8 items, and the received social support scale includes 15 items. People responded to items on a 5-point scale, ranging from 1 (“definitely untrue”) to 5 (“definitely true”). Cronbach’s alpha coefficients for the present study were 0.81 for perceived social support and 0.84 for received social support.

#### Meaning-making

Meaning-making was measured using the Meaning-Making Questionnaire (MMQ) by Krok and Zarzycka [45]. The MMQ evaluates one’s cognitive capacity to comprehend and assimilate challenging or ambiguous life events into coherent structures of personal meaning, beliefs, and goals. It includes 8 items that are rated on a 5-point scale, ranging from 1 (“never”) to 5 (“very often”). Higher scores represent a more active process of meaning-making. The Cronbach’s alpha coefficient for the present study was 0.86.

#### Fear of recurrence

The Cancer Worry Scale by Custers et al. was used to assess fear of recurrence which is conceptualized as worry about the likelihood of cancer’s recurrence and its interference with daily functioning [46]. The scale was adapted and validated in Polish by Krok and Telka [47]. This widely used scale contains 8 items that are rated on a 4-point scale, ranging from 1 (“never”) to 4 (“almost always”). Higher scores represent more frequent worries about cancer (i.e., a greater fear of cancer recurrence). The Cronbach’s alpha coefficient for the present study was 0.89.

#### Illness acceptance

The Acceptance of Life with the Disease Scale by Janowski et al. was utilized to assess participants’ adaptation to the disease in terms of the ability to accept health conditions and maintain overall life satisfaction [48]. The scale contains 20 items that are rated on a 5-point scale, ranging from 1 (“no”) to 5 (“yes”). It has three subscales: (a) satisfaction with life, (b) reconciliation with the disease, and (c) self-distancing from the disease. The total score, which is computed by adding the three subscales, was used in the current study. Higher scores indicate a higher level of illness acceptance. The Cronbach’s alpha coefficient was 0.90.

### Data Analysis

All statistical analyses were conducted with SPSS (version 28) software. First, we performed a priori power analysis G* to calculate a sufficient sample size. The following parameters were applied: 90% power (1 − β), α = 0.05 [49]. The results showed that a total of 236 participants was required for the study. Next, two-tailed correlations were calculated among all the variables. Finally, serial mediation analysis (Model 6) was used to test the direct and indirect effects between the variables [50]. The bootstrapping procedure (5,000 samples; 95% confidence intervals [CI]) was applied, so the size of our sample (*n* = 100 or more) precludes type II errors. We ran our mediation analysis for two models with perceived and received social support examined separately as two independent variables. Therefore, we calculated separate mediation effects for meaning-making and fear of recurrence under: Model 1 for perceived social support and Model 2 for received social support. Meaning-making and fear of recurrence were entered as serial mediators, so indirect effects were calculated for them separately (i.e., for each mediator: Perceived social support—Meaning-making—Illness acceptance) and jointly (i.e., for both mediators: Perceived social support—Meaning-making—Fear of recurrence—Illness acceptance). In addition, missing data were handled by using the method of case-wise mean substitution that imputes average scores from the corresponding subscale. The level of missing data in our study was very low, i.e., around 1%, so it was acceptable to use this procedure.

## Results

### Descriptive Statistics and Correlational Analysis

The participants were characterized by the following demographic and medical data: (a) employment status—full-time/part-time work: *N* = 121, 49.1%; retired/domestic work: *N* = 125, 50.9%, (b) marital status—married/domestic partner: *N* = 182, 73.9%; single/widowed: *N* = 64, 26.1%, (c) body mass index: *M* = 22.36, *SD* = 2.37, and (d) illness duration: *M* = 3.59, *SD* = 4.51. First, we calculated descriptive statistics for all the variables in our sample (Table 1). In all the cases, the mean scores were above the scale midpoint, which was 2.5. Initial correlation analysis demonstrated that all the Pearson coefficients turned out to be statistically significant. Both perceived and received social support were positively associated with meaning-making and illness acceptance, yet they were negatively associated with fear of recurrence. In addition, meaning-making was negatively correlated with fear of recurrence.

**Table 1:** Means, Standard Deviations, and Correlations Among Perceived and Received Social Support, Meaning-Making, Fear of Recurrence, and Illness Acceptance

Variables	*M*	*SD*	Skewness	Kurtosis	Scores range	1	2	3	4
1. Perceived social support	3.42	0.54	0.13	0.24	1–5	–			
2. Received social support	3.32	0.46	−0.01	0.61	1–5	0.56***	–		
3. Meaning-making	2.95	0.96	0.21	−0.74	1–5	0.35***	0.30***	–	
4. Fear of recurrence	2.61	0.60	0.10	−0.54	1–5	−0.38***	−0.23***	−0.27***	
5. Illness acceptance	3.38	0.71	−0.71	0.39	1–5	0.36***	0.31***	0.49***	−0.33***

*p < .05; **p < .01; ***p < .001.

### Mediation Analysis

To investigate whether meaning-making and fear of recurrence would serially mediate the relationship of perceived and received social support with illness acceptance, mediation analysis (Model 6) with the bootstrapping procedure (sample = 5,000; 95% bias-corrected CIs) suggested by Hayes [50] was applied to calculate the mediational effects (i.e., standardized parameter estimates). The results are given in Table 2.

**Table 2:** Mediation Estimates for Meaning-Making and Fear of Recurrence in the Relationship of Perceived and Received Social Support With Illness Acceptance (Standardized Parameter Estimates)

Variables	β	*SE*	*t*	Model *R*^2^
Direct effects				
Perceived social support as Independent Variable				
Perceived social support—Meaning-making	0.35	0.11	5.90***	0.13***
Perceived social support—Fear of recurrence	−0.32	0.11	−5.21***	
Meaning-making—Fear of recurrence	−0.15	0.06	−2.48*	0.16***
Meaning-making—Illness acceptance	0.40	0.06	7.05***	
Fear of recurrence—Illness acceptance	−0.16	0.05	−2.68**	
Perceived social support—Illness acceptance	0.16	0.10	2.67**	0.31***
Received social support as Dependent Variable				
Received social support—Meaning-making	0.30	0.12	4.99***	.09***
Received social support—Fear of recurrence	−0.16	0.12	−2.56*	
Meaning-making—Fear of recurrence	−0.22	0.06	−3.45**	0.10***
Meaning-making—Illness acceptance	0.41	0.06	7.20***	
Fear of recurrence—Illness acceptance	−0.18	0.06	−3.26**	
Received social support—Illness acceptance	0.14	0.12	2.51*	0.31***
Indirect effects	*Effect*	*SE*	*LLCI*	*ULCI*
Perceived social support—Meaning-making—Illness acceptance	0.14	0.03	0.08	0.21
Perceived social support—Fear of recurrence—Illness acceptance	0.05	0.02	0.02	0.10
Perceived social support—Meaning-making—Fear of recurrence—Illness acceptance	0.20	0.03	0.13	0.28
Received social support—Meaning-making—Illness acceptance	0.12	0.03	0.06	0.20
Received social support—Fear of recurrence—Illness acceptance	0.03	0.02	0.01	0.06
Received social support—Meaning-making—Fear of recurrence—Illness acceptance	0.17	0.04	0.09	0.24
Total effect				
Perceived social support—Illness acceptance	0.36	0.11	6.10***	0.13***
Received social support—Illness acceptance	0.31	0.12	5.11***	0.10***

*p < .05, **p < .01, ***p < .001.

The examination of direct effects revealed that perceived social support was positively related to meaning-making, but negatively related to fear of recurrence. Meaning-making was also negatively related to fear of recurrence. With regard to illness acceptance, meaning-making, and perceived social support were positively related to it, whereas fear of recurrence was negatively related. The total indirect effect showed that meaning-making and fear of recurrence were serial mediators between perceived social support and illness acceptance. Taking into account the separate mediational effects, both meaning-making and fear of recurrence mediated relationships between perceived social support and illness acceptance.

Next, received social support was positively associated with meaning-making but negatively associated with fear of recurrence. Meaning-making was also negatively associated with fear of recurrence. Furthermore, meaning-making and received social support were positively related to illness acceptance, while fear of recurrence had a negative relation with it. The examination of indirect effects indicated that meaning-making and fear of recurrence were serial mediators between received social support and illness acceptance. Regarding their separate mediational effects, both meaning-making and fear of recurrence mediated relationships between received social support and illness acceptance. Total effects of perceived and received social support on illness acceptance turned out to be significant, respectively.

**Fig. 2.**
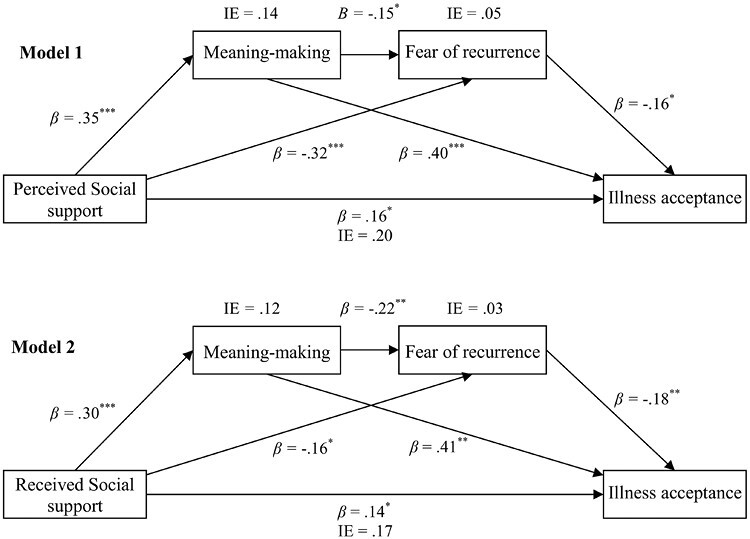
The final mediation model of meaning-making and fear of recurrence in associations between perceived and received social support and illness acceptance, respectively, Model 1 and Model 2 (standardized coefficients β, and indirect effects IE)

Finally, we decided to evaluate the separate mediation effects of meaning-making and fear of recurrence by using effect-contrast procedures. For perceived social support, the results showed differences in the mediating powers of meaning-making and fear of recurrence (Indirect Effect = 0.09; CIs [0.01,0.18]), with the first having a stronger mediation effect than the latter. For received social support, there were also significant differences in the mediating powers of meaning-making versus fear of recurrence (Indirect Effect = 0.10; CIs [0.03,0.17]); the former exerted a stronger mediation effect than the latter (Fig. 2).

## Discussion and Conclusion

### Discussion

The aim of the present study was to verify whether meaning-making and fear of recurrence are serial mediators in the relationship of perceived and received social support with illness acceptance among breast cancer patients. In general, our results suggest that while meaning-making and fear of recurrence were serial mediators between perceived and received social support and illness acceptance, respectively, their mediating powers were different.

#### Relationships between social support and meaning-making and fear of recurrence

The result that both perceived and received social support were positively related to meaning-making, but negatively related to fear of recurrence is consistent with our first hypothesis as well as previous findings relating the importance of supportive social relations to meaning-making processes [15, 26]. Women with breast cancer who realize the availability of supportive actions and actually receive them are characterized by a more efficient ability to comprehend and integrate challenging life events and experience less fear and anxiety related to potential relapse of cancer than those that do not. However, while other studies found a stronger effect for perceived social support than received social support [51], in the current study the strength of these relationships was comparable. We extended those findings by showing that both perceived and received social support play a significant role in cancer patients functioning.

This may be a consequence of a relatively high level of anxiety and fear experienced by women cancer patients [39]. According to Cicero et al. [40], perceived social support from others may predict the patient’s tendency to consider cancer as a challenge and consequently take an active role in thinking about the illness in constructive terms. In addition, patients who are not lonely and experience social support report a lower fear of recurrence [31, 32]. This might stem from the fact that more frequent social contacts, which help patients not think about the disease and their future all the time, provide a sense of availability of potential support in the future.

#### Associations of meaning-making and fear of recurrence with illness acceptance

In line with our second hypothesis, both meaning-making and fear of recurrence were significant predictors of illness acceptance. However, while meaning-making was positively related to illness acceptance, fear of recurrence had a negative relation. This finding is consistent with previous research demonstrating that cancer patients who managed to construct a coherent vision of life during traumatic and potentially destructive incidents are characterized by better adjustment [9, 25, 52], higher well-being [25, 45], and general anxiety [26]. At the same time, the result regarding the negative association between fear of recurrence and illness acceptance confirms previous studies in which cancer patients who experienced worries and fear reported a relatively low quality of life [30] and adjustment to illness [39]. Yet, our finding extends previous findings by indicating that both meaning- and fear-related processes need to be taken into account while dealing with consequences of cancer. Searching for meaning in life through programs such as those created by Rębiałkowska-Stankiewicz not only help participants create new meaning but also may be very useful, because talking openly about the disease reduces fear [53].

A potential explanation for these results may lie in the fact that the ability to form a meaningful perspective may represent a resource for women confronted with breast cancer who experience meaning threats regarding their health, body image, or personal relations. For instance, mothers could choose to think about their children and their future in a positive light, which helps them to remain strong and accept their cancer. Furthermore, the idea of living for children can prompt women to accept a burdensome therapy [54]. This view is supported by meta-analyses that confirm positive correlations between meaning in life and acceptance of cancer [55]. On the other hand, fear of recurrence seems to lower the ability to accept the potential detrimental consequences of breast cancer, which makes the process of accepting the illness more difficult. Similar effects were found in studies in which fear of recurrence was related to depressive and anxiety symptoms in breast cancer survivors [38].

It is also worth emphasizing that the effect-contrast procedures showed that the mediating power of meaning-making was more significant than fear of recurrence. Perhaps because an effective meaning-making process has more long-term effects, reformulating the meaning of cancer and important goals can provide a stable basis and sense of emotional support for the patient. This is confirmed by a study in which cancer survivors who succeeded in meaning-making efforts experienced relatively more meaning in life than before the diagnosis [25]. In addition, since acceptance of illness has been conceptualized as a dynamic process of emotion-focused coping [17], breast cancer patients can also use acceptance coping that comprises relying on self-compassion or communicating their own feelings about the illness to other people. This activity may result in reduced emotional reactivity (i.e., less fear of recurrence), reappraisal of cancer-related stressors (i.e., meaning-making processes), and significant improvement in emotional well-being (i.e., higher acceptance of illness).

#### Meaning-making and fear of recurrence as serial mediators

The main finding of this study is a serial mediation of meaning-making and fear of recurrence in the relationship of both perceived and received social support with illness acceptance, which confirms our third hypothesis. This result seems interesting as it justifies investigating meaning-making and fear of recurrence as *factors operating in concerted action*. Therefore, it sheds new light on previous research regarding the relationship of social support with adjustment to illness [16, 22] by demonstrating a concrete function of meaning and fear. Although they are independent psychological constructs, examining both in relation to illness acceptance provides more information than investigating either alone. From a psychological point of view, their co-occurrence seems justified, as people who are able to develop a sense of meaning and purpose and are optimistic about the future will experience lower anxiety about a potential relapse of the disease than those who do not [37]. This can consequently translate into a relatively higher level of illness acceptance.

The serial mediation function of meaning-making and fear of recurrence can be understood within Park’s [28, 35] meaning-making model, according to which cancer symptoms challenge people’s beliefs and goals, generating distress which, in turn, activates a meaning-making process. In this context, perceived and received social support can be an important source of mental strength and motivation to make meaning. Individuals try to reduce their distress by finding meaningful views and goals through cognitive activities that decrease their fear of future detrimental consequences and potential relapses of cancer [51]. This enables cancer patients to constructively reorganize their lives on the basis of new meanings and goals, which are formulated in response to perceived and received forms of social support. Therefore, the serial mediation effects demonstrated two significant things. First, the interplay of meaning-making and fear of recurrence determines the extent to which breast cancer patients are able to accept the negative consequences of their illness. Second, the prerequisite for a positive attitude of acceptance is the attainment of constructive meanings and goals and the optimal reduction/management of the fear of recurrence as much as possible. As a consequence, breast cancer patients can come to understand the illness in a different, more optimistic way and reorganize their beliefs and goals in order to restore inner cognitive and emotional stability. This interpretation is supported by the finding obtained by Pasek et al. [16] in which sense of coherence (i.e., the construct including structures of meaning) mediated the relationship between perceived social support and illness acceptance in cancer patients.

#### Limitations

The present study is not without limitations. First, as our results emerge from a cross-sectional study design, the mediational model does not allow us to draw any causal conclusions regarding the observed relationships. Longitudinal research, especially with a meaning-making intervention among patients with different levels of social support, would be needed to determine causality. Second, the method we used to measure meaning-making in this study may be questioned. Even though the MMQ [45] is a recognized and empirically verified tool, meaning-making is a dynamic process that might require assessment at different stages of a disease. Third, our study only considered perceived and received social support. Different kinds of social support could play different roles in breast cancer coping, like the actual frequency of social contacts and their quality, financial support, or the perceived or real adequacy of social supports. Finally, as our study was conducted in Poland, the results may not be fully generalizable to other countries in Western Europe or the USA, as they have a different clinical or medical system (e.g., specific cancer prevention systems, more diverse approaches to psychosomatic interventions or the use of radiotherapy/chemotherapy treatment interventions), resulting in differences in the mental experience of breast cancer patients.

### Conclusions

In sum, despite the above limitations, the current study has shed new light on the mediational model with a serial mediation of meaning-making and fear of recurrence in the relationship of both perceived and received social support with breast cancer and perhaps general, serious illness acceptance. Notably, our analysis showed that the mediating power of meaning-making is more significant than fear of recurrence. It suggests that creating an intervention that combines providing aid in enhancing meaning-making and lowering fear of recurrence might benefit women with breast cancer.

### Implications for Practice

The novelty of this research is the identification of meaning-making and fear of recurrence as important clinical factors that underlie the relationship of social support with illness acceptance among patients with breast cancer. By focusing on the sphere of meaning and purpose, care for patients with breast cancer can become more effective. In addition, strategies aimed at decreasing fear of recurrence will have an impact not only on effective coping with the illness, but also on the quality of social support offered to the patients. Furthermore, the mediating function of meaning-making and fear of recurrence should be acknowledged and encouraged in the process of providing medical care to patients with breast cancer. By increasing the knowledge of intrapersonal cognitive (meaning-making) and emotional (fear and anxiety) processes, the results of this study may contribute to the implementation of treatment programs that provide cancer patients with adequate and effective forms of social support.
